# What is the cause of *P*-wave undersensing in this CRT-D device?

**DOI:** 10.1007/s12471-018-1130-4

**Published:** 2018-06-26

**Authors:** J. Schroemges, F. A. L. E. Bracke, B. M. van Gelder

**Affiliations:** Department of Electrophysiology, Catharina Hospital, Eindhoven, The Netherlands

In a 70-year-old male suffering from coronary artery disease in New York Heart Association class II–III with an ejection fraction of 25%, a DDDR pacemaker was replaced by a CRT-D device. A Medtronic Claria MRI Quad was implanted in January 2017.

Recently the patient presented at the pacemaker clinic with reduced exercise tolerance and shortness of breath even after the short walk from the parking lot to the pacemaker clinic. The ECG recorded during these complaints is presented in Fig. 1 and shows *P*-wave undersensing, which normalised after a few minutes of rest. *P*-wave undersensing could easily be evoked again by the patient repeat exercising for a few minutes.

**Fig. 1:**
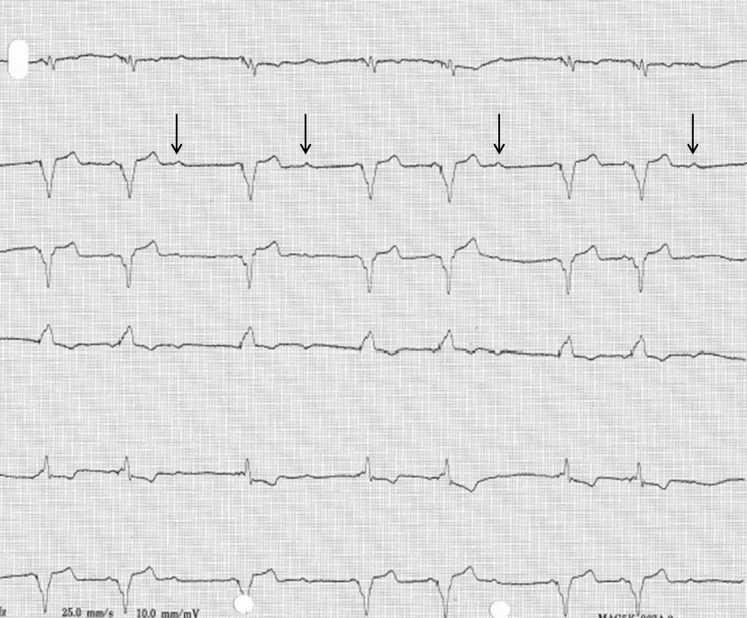
*From top to bottom*: ECG leads I, II, III, aVR, aVL, aVF, showing atrial synchronous ventricular pacing with intermittent *P*-wave undersensing (*arrows*)

What was the cause of *P*-wave undersensing in this device?

## Answer

You will find the answer elsewhere in this issue.

